# Effect of auricular point pressing therapy on postoperative pain of fracture: A protocol for systematic review and meta-analysis

**DOI:** 10.1097/MD.0000000000023696

**Published:** 2020-12-18

**Authors:** Zhe Yin, Wenjun Zhang, Yi Zeng, Xi Su

**Affiliations:** a Key Laboratory for Biorheological Science and Technology of Ministry of Education (Chongqing University), Chongqing University Cancer Hospital, Chongqing; b Department of Community Family Health, Maternaland Child Health Hospital of Hubei Province, Wuhan, Hubei Province, China.

**Keywords:** auricular acupoint pressing, meta-analysis, postoperative pain in fracture, protocol

## Abstract

**Background:**

In clinical practices, postoperative fracture patients are often treated with analgesics. As one of the alternative therapies for nondrug analgesia, auricular point pressing has advantages of simple operation, easy to use, no injury and adverse reactions, and great potential for development. In this study, the effect of auricular point pressing therapy on postoperative pain of fracture was objectively evaluated through the method of meta-analysis, so as to provide evidence for clinical applications.

**Methods:**

PubMed, Web of Science, Cochrane Library, EMBASE, Wan fang Database, Chinese Scientific Journal Database, China National Knowledge Infrastructure Database, and Chinese Biomedical Literature Database were systematically searched and randomized controlled trials on auricular point pressing in the treatment of postoperative pain after fracture were includes. After independent literature screening, data extraction and quality evaluation by 2 researchers, the original data was retrieved, merged, and analyzed. RevMan 5.3 software was adopted for meta-analysis

**Results:**

This study could provide high-quality evidence to evaluate the effect of auricular point pressing therapy on postoperative pain of fracture.

**Conclusion:**

This systematic review explored whether auricular point pressing therapy is effective on the intervention of postoperative pain after fracture.

**OSF Registration Number:**

DOI 10.17605/OSF.IO/AZ4JQ.

## 1 Introduction

The postoperative pain of fracture mainly is a certain degree of acute pain, and is a complex physiological reaction caused by body itself and surgical trauma.^[1]^ Pain has become the fifth vital sign, followed by body temperature, pulse, breathing, and blood pressure.^[2]^ At the same time, the pain after fracture can promote the release of various endogenous substances in human bodies, act on skeletal muscle, myocardial or vascular smooth muscle, increase peripheral circulation resistance, and produce adverse reactions such as elevated blood pressure and accelerated heart rhythm.^[3]^ Postoperative fracture patients often take analgesics to relieve pain. Effective postoperative analgesia can reduce the secretion of catecholamine in patients, inhibit stress reaction, and contribute to the rehabilitation of patients.^[4]^ However, improper administration of analgesics may cause adverse reactions such as gastrointestinal discomfort, liver and kidney function damage, cardiovascular system damage, and so on.^[5–7]^

Auricular point therapy of Traditional Chinese Medicine (TCM) is one of the external therapies with the characteristics of TCM, including pressing, acupuncture, bloodletting, needle imbedding, Tuina, and other operations.^[8–10]^ According to TCM, auricular points are specific acupoints distributed on the auricle of human body, and they are the reaction points of pathological changes of Zang-fu Organs and meridians and collaterals on body surface.^[11,12]^ According to bioelectricity theory, the abnormal bioelectricity in pathological changes of the human body can cause the decrease of the resistance of corresponding auricular points. Related studies proved that after, 1 to 2 weeks of applying voltage to the auricle skin of peritonitis rats, the low resistance state of the skin around auricle improved.^[13]^

Clinically, auricular point pressing is the most commonly applied auricular point stimulation method at present, with advantages of long-lasting effect, simple operation, and less side effects.^[14]^ Clinical reports proved that this therapy plays a positive role in the intervention of headache, insomnia, and neurasthenia.^[15–17]^ At present, the effect of auricular point pressing therapy on postoperative pain of fracture lakes meta-analysis, thus hindering the spread of evidence. Therefore, according to the objective clinical needs, this study conducted a systematic review and meta-analysis of randomized controlled trials (RCTs) on the effect of auricular point pressing therapy on postoperative pain after fracture, so as to provide support for the spread of evidence.

## 2 Methods

### 2.1 Study registration

The protocol of this review was registered in OSF (OSF registration number: DOI 10.17605/OSF.IO/AZ4JQ). It was reported to follow the statement guidelines of preferred reporting items for systematic reviews and meta-analyses protocol.^[18]^

### 2.2 Inclusion criteria for study selection

#### 2.2.1 Types of studies

A RCTs was enrolled to investigate the effects of auricular plaster therapy on postoperative pain after fracture surgery published in Chinese and English.

Non-RCTs, cohort studies, case reports, experimental studies, the data of the included study are missed or incomplete, and duplicate publications were excluded

#### 2.2.2 Types of participants

Regardless of nationality, race, gender, occupation, and educational background, patients after fracture surgery were included. Patients with pain caused by tumors, nervous system diseases, and other causes were excluded.

#### 2.2.3 Types of interventions

The experimental group was treated with auricular point pressing therapy. The control group was treated with placebo, drugs, or other alternative therapy.

#### 2.2.4 Types of outcome measures

##### 2.2.4.1 Primary outcomes

Postoperative visual pain simulation score.

##### 2.2.4.2 Secondary outcomes

1) Adverse reactions.
2) Number of pain outbreaks.
3) Patient satisfaction.

### 2.3 Data sources

PubMed, Web of Science, Cochrane Library, EMBASE, Wan fang Database, Chinese Scientific Journal Database, China National Knowledge Infrastructure Database, and Chinese Biomedical Literature Database were systematically searched.

### 2.4 Searching strategy

The details of PubMed's search strategy are illustrated in Table 1, including all search terms, while similar search strategies are applied to other electronic databases.

**Table 1:** Search strategy in PubMed database.

Number	Search terms
#1	Fractures[Title/Abstract]
#2	Fracture[Title/Abstract]
#3	or/1–2
#4	Auricular[Title/Abstract]
#5	Point[Title/Abstract]
#6	Points[Title/Abstract]
#7	or/5–6
#8	#3 and #4 and #7

### 2.5 Data collection and analysis

#### 2.5.1 Selection of studies

**Figure 1:**
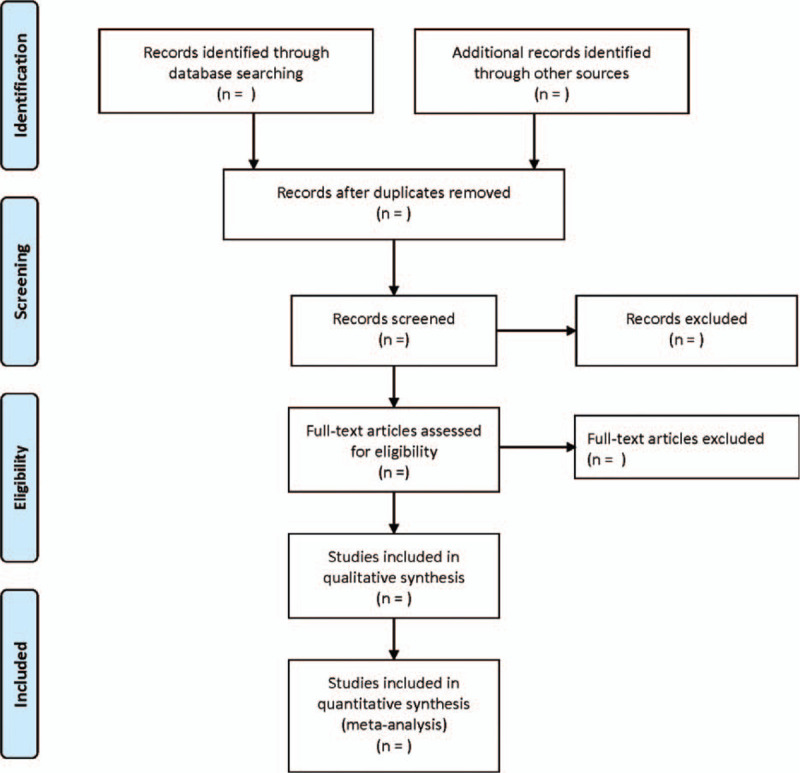
Flow diagram of study selection process.

Two researchers independently carried out literature screening, data extraction and cross-checking, and dissenting literatures or data were decided by the third researcher. According to the selection and exclusion criteria, the literature screening and data extraction were conducted strictly, and the whole screening and extraction process was recorded. The screening flow chart of this study is demonstrated in Figure 1.

#### 2.5.2 Data extraction and management

Two independent researchers extracted data from qualified literatures and filled out data collection forms. Any objection would be resolved by consensus or consultation with a third researcher. Excel 2013 was used for data management. We extracted the following data: title, first author, year of publication, country, journal, age, sex, sample size, inclusion and exclusion criteria, baseline, intervention details, follow-up time, outcome indicators, and bias risk.

#### 2.5.3 Assessment of risk of bias

The quality evaluation of literatures is based on Cochrane handbook system evaluation manual. The quality evaluation items include:

(1) Is the allocation method random?
(2) Is it hidden?
(3) Whether to adopt the blind method, or not?
(4) Is there any incomplete data?
(5) Is there selective bias?
(6) Are there any other biases?

According to the criteria, the included studies were divided into 3 levels: low, medium, and high.

#### 2.5.4 Measures of treatment effect

Standard mean difference with 95% confidence intervals. was used as continuous data, and the dichotomous outcomes were estimated by the risk ratio with 95% confidence intervals.

#### 2.5.5 Management of missing data

If any data is missing, requesting the original data by email. If the missing data cannot be obtained, the data could be excluded from the study.

#### 2.5.6 Assessment of heterogeneity and data synthesis

Heterogeneity test: *Q* test was applied to qualitatively determine inter-study heterogeneity: If *P* ≥ .1, there is no inter-study heterogeneity, and if *P* < .1, there is inter-study heterogeneity. Meanwhile, *I*^2^ value is adopted to quantitatively evaluate the inter-study heterogeneity: If *I*^2^ ≤ 50%, the heterogeneity is considered to be good, and the fixed-effect model would be adopted. If *I*^2^ > 50%, it indicates significant heterogeneity, and the source of heterogeneity would be explored through subgroup analysis or sensitivity analysis. If there is no obvious clinical or methodological heterogeneity, it would be considered as statistical heterogeneity, and the random-effect model would be applied for analysis. Descriptive analysis would be used if there is significant clinical heterogeneity between the 2 groups, and subgroup analysis is not required.

#### 2.5.7 Assessment of reporting biases

If more than 10 studies are included, a funnel chart would be utilized to assess the report bias.^[19–21]^ In addition, publication bias was further quantitatively evaluated by Egger and Begger test.

#### 2.5.8 Subgroup analysis

When heterogeneity is discovered (such as different types of treatment, different types of fractures, patient age, and publication year), subgroup analysis would be applied to find out the source of heterogeneity.

#### 2.5.9 Sensitivity analysis

Through the study of large weight of elimination effect, the sensitivity analysis was performed to test the stability of the results of meta-analysis.

#### 2.5.10 Ethics and dissemination

The content of this article does not involve moral approval or ethical review and would be presented in print or at relevant conferences.

## 3 Discussion

Clinically, patients after fracture surgery are often treated with analgesics, including opioid analgesics and nonsteroidal anti-inflammatory drugs. Improper administration of analgesics may cause adverse reactions such as tolerance, dependence, toxic reactions, mental reactions, gastrointestinal reactions, liver and kidney function damage, cardiovascular system damage, and so on.^[22,23]^

With the change of modern medical model, orthopedic patients have higher requirements for nursing, and patients and medical staff are no longer satisfied with simple fracture healing. Instead, they pursue a series of physical and mental therapeutic effects, including pain management, functional rehabilitation, and orthopedic care.^[24]^ Among them, the mode of pain management changed from a single mode with anesthesiologists as the main body to a combined mode with nurses as the main body. Auricular plaster therapy in TCM is a traditional and common therapy for nondrug analgesia.^[25]^ It is easy to operate, without injury and rare adverse reactions.^[26]^ Although many clinical studies confirmed that auricular point pressing therapy is effective for postoperative pain after fracture, its efficacy and safety are still controversial.

The purpose of this study was to systematically review and evaluate all RCTs of the effect of auricular point pressing therapy on postoperative pain after fracture. The systematic review and meta-analysis of this article provide convincing conclusions for the efficacy and safety of auricular point pressing therapy for the treatment of postoperative pain after fracture. In addition, this study helps clinical doctors and nurses to intervene postoperative pain of fracture, benefit patients, and provide reliable reference for its wide application.

## Author contributions

**Conceptualization:** Wenjun Zhang.

**Data curation:** Wenjun Zhang.

**Formal analysis:** Wenjun Zhang, Yi Zeng.

**Investigation:** Yi Zeng.

**Methodology:** Yi Zeng.

**Project administration:** Xi Su, Zhe Yin.

**Resources:** Yin Zhe, Yi Zeng.

**Software:** Zhe Yin, Yi Zeng.

**Supervision:** Xi Su.

**Validation:** Xi Su.

**Visualization and software:** Zhe Yin.

**Visualization:** Zhe Yin.

**Writing – original draft:** Zhe Yin and Xi Su.

**Writing – review & editing:** Zhe Yin and Xi Su.

## References

[1] Yamamoto N Sakura S Noda T Comparison of the postoperative analgesic efficacies of intravenous acetaminophen and fascia iliaca compartment block in hip fracture surgery: a randomised controlled trial. Injury 2019;50:1689–93. 30904248 10.1016/j.injury.2019.03.008

[2] Torma L Pain--the fifth vital sign. Pulse 1999;36:16. 10614473

[3] Pasyar N Rambod M Kahkhaee FR The effect of foot massage on pain intensity and anxiety in patients having undergone a tibial shaft fracture surgery: a randomized clinical trial. J Orthop Trauma 2018;32:e482–6. 30444801 10.1097/BOT.0000000000001320

[4] Sunadi A Ifadah E Syarif MNO The effect of deep breathing relaxation to reduce post operative pain in lower limb fracture. Enferm Clin 2020;30: Suppl 3: 143–5.

[5] Effectiveness of multimodal pain therapy on reducing opioid use in surgical geriatric hip fracture patients. J Trauma Nurs 2020;27:E13–e14. 32658062 10.1097/JTN.0000000000000522

[6] Unneby A Svensson PO Gustafson PY Complications with focus on delirium during hospital stay related to femoral nerve block compared to conventional pain management among patients with hip fracture - a randomised controlled trial. Injury 2020;51:1634–41. 32360090 10.1016/j.injury.2020.04.013

[7] Schulte SS Fernandez I Van Tienderen R Impact of the fascia iliaca block on pain, opioid consumption, and ambulation for patients with hip fractures: a prospective, randomized study. J Orthop Trauma 2020;34:533–8. 32358477 10.1097/BOT.0000000000001795

[8] Han Q Yang L Huang SY Effectiveness of auricular point therapy for cancer-related fatigue: a systematic review and meta-analysis. J Adv Nurs 2020;00:1–2. 10.1111/jan.14375 32428970

[9] Yang Y Wen J Hong J The effects of auricular therapy for cancer pain: a systematic review and meta-analysis. Evid Based Complement Alternat Med: eCAM 2020;2020:1618767. 32565846 10.1155/2020/1618767 PMC7267873

[10] Gao J Chen G He H The effect of auricular therapy on blood pressure: a systematic review and meta-analysis. Eur J Cardiovasc Nurs 2020;19:20–30. 31583887 10.1177/1474515119876778 PMC6927068

[11] Liu M Tong Y Chai L Effects of auricular point acupressure on pain relief: a systematic review. Pain Manag Nurs 2020;20:17–24. 10.1016/j.pmn.2020.07.007 32950391

[12] Yeh CH Li C Glick R A prospective randomized controlled study of auricular point acupressure to manage chronic low back pain in older adults: study protocol. Trials 2020;21:99. 31959226 10.1186/s13063-019-4016-x PMC6972012

[13] Kawakita K Kawamura H Keino H Development of the low impedance points in the auricular skin of experimental peritonitis rats. Am J Chin Med 1991;19:199–205. 1767791 10.1142/S0192415X91000272

[14] Yu J Li QZ Clinical observation of auricular acupoint therapy for pain in early-stage extremity trauma. J Acupunct Tuina Sci 2017;15:219–22.

[15] Ye YF Mei R Ren JX Intervention of auricular point sticking on perioperative psychological stress in patients with anorectal diseases. Zhongguo Zhen Jiu = Chin Acupunct Moxib 2019;39:605–8. 10.13703/j.0255-2930.2019.06.010 31190496

[16] Kuo HC Tsao Y Tu HY Pilot randomized controlled trial of auricular point acupressure for sleep disturbances in women with ovarian cancer. Res Nurs Health 2018;41:469–79. 30024027 10.1002/nur.21885

[17] Ko YL Lin SC Lin PC Effect of auricular acupressure for postpartum insomnia: an uncontrolled clinical trial. J Clin Nurs 2016;25:332–9. 26612319 10.1111/jocn.13053

[18] Shamseer L Moher D Clarke M Preferred reporting items for systematic review and meta-analysis protocols (PRISMA-P) 2015: elaboration and explanation. BMJ (Clin Res Ed) 2015;350:g7647. 10.1136/bmj.g7647 25555855

[19] Lewis SJ Zammit S Gunnell D Bias in meta-analysis detected by a simple, graphical test. BMJ Clin Res 1997;315:629–34. 10.1136/bmj.315.7109.629 PMC2127453 9310563

[20] Duval S Tweedie R Trim and fill: a simple funnel-plot-based method of testing and adjusting for publication bias in meta-analysis. Biometrics 2000;56:455–63. 10877304 10.1111/j.0006-341x.2000.00455.x

[21] Zhang Q Jin Y Li X Plasminogen activator inhibitor-1 (PAI-1) 4G/5G promoter polymorphisms and risk of venous thromboembolism – a meta-analysis and systematic review. VASA Zeitschrift fur Gefasskrankheiten 2020;49:141–6. 31920171 10.1024/0301-1526/a000839

[22] Bossart M Becker M Hadji P Compliance, analgesic use and side-effect protection within a German cohort of the TEAM trial. Anticancer Res 2012;32:3933–8. 22993340

[23] Benyamin R Trescot AM Datta S Opioid complications and side effects. Pain Physician 2008;11: 2 Suppl: 105–20. 18443635

[24] Elsevier H Cannada LK Management of pain associated with fractures. Curr Osteoporos Rep 2020;18:130–7. 32236905 10.1007/s11914-020-00578-3

[25] Yeh CH Chien LC Chiang YC Reduction in nausea and vomiting in children undergoing cancer chemotherapy by either appropriate or sham auricular acupuncture points with standard care. J Altern Complement Med (New York, NY) 2012;18:334–40. 10.1089/acm.2011.0102 22515794

[26] Li WS Cui SS Li WY Effects of magnetic auricular point-sticking on adjuvant anesthesia and postoperative recovery of body function. Chin Acupunct Moxib 2011;31:349–52. 21528603

